# Supplementary material for: CD74 supports accumulation and function of regulatory T cells in tumors
Source: Nat Commun. 2024 May 3;15:3749. doi: 10.1038/s41467-024-47981-3 (PMC11068745; doi:10.1038/s41467-024-47981-3)
Supplement: Supplementary file 1 — Supplementary information [file 41467_2024_47981_MOESM1_ESM.pdf]

Supplementary information for

CD74 supports accumulation and function of regulatory T cells in tumors

Authors

Elisa Bonnin[1,2], Maria Rodrigo Riestra[1,2], Federico Marziali[1,2], Rafael Mena Osuna[1,2], Jordan Denizeau[1,2], Mathieu Maurin[1], Juan Jose Saez[1], Mabel Jouve[1], Pierre-Emmanuel Bonté[1], Wilfrid Richer[1,2], Fabien Nevo[3], Sebastien Lemoine[3], Nicolas Girard[1,4,5], Marine Lefevre[6], Edith Borcoman[7], Anne Vincent-Salomon[5,8], Sylvain Baulande[9], Helene D. Moreau[1], Christine Sedlik[1,2], Claire Hivroz[1], Ana-Maria Lennon-Duménil[1], Jimena Tosello Boari[1,2,#], Eliane Piaggio[1,2,3,#]

The PDF file includes:

Supplementary Figures (Fig. S#)

Fig.S1: CD74 is overexpressed in Tregs from different tumor types compared to Tconvs and CD8+ cells.
Fig.S2: Phenotypic characterization of WT and CD74KO Tregs.
Fig.S3: Description of T cells in NSG mice transferred with PBMCs alone or together with WT or CD74KO expTregs.
Fig.S4: Repartition of T cells in tumor-bearing mice transferred with PBMCs alone or together with WT expTregs or CD74KO expTregs.

Fig.S5: Tumor invasion, MIF independency, velocity and trajectory of WT and CD74KO Tregs.

FigS6: Transcriptomic analysis of CD74KO and WT Tregs.
Fig.S7: Perturbed cell shape, organelle organization and cell interaction of CD74KO Tregs.
Fig.S8: Generation and characterization of Foxp3-specific CD74 conditional knock-out mice (CD74cKO mice).

Supplementary Tables (TableS#)

Table-S1. List of antibodies and markers used for cytometry and immunohistochemistry.
Table-S2. Description of the different stimulation implement to challenge Tregs.

Figure.S1

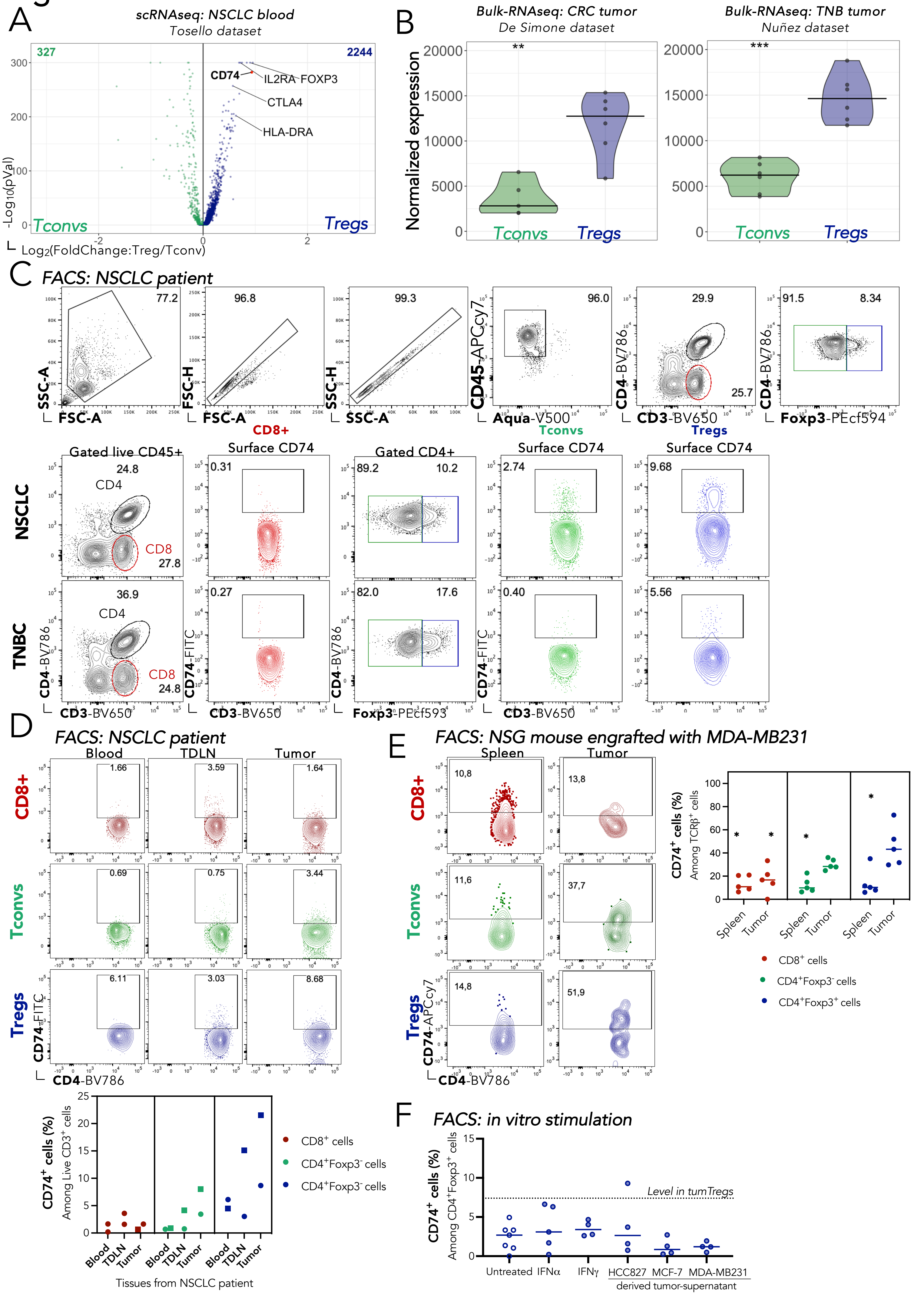
A
scRNAseq: NSCLC blood
Tosello dataset
327
2244
CD74
IL2RA FOXP3
CTLA4
HLA-DRA
-Log10(pVal)
Tconvs
Tregs
Log2(FoldChange:Treg/Tconv)
B
Bulk-RNAseq: CRC tumor
De Simone dataset
Bulk-RNAseq: TNB tumor
Nuñez dataset
Normalized expression
**
***
Tconvs
Tregs
C
FACS: NSCLC patient
77.2
96.8
99.3
96.0
29.9
25.7
91.5
8.34
SSC-A
FSC-A
FSC-H
SSC-H
CD45-APCcy7
Aqua-V500
CD4-BV786
CD3-BV650
Foxp3-PEcf594
CD8+
Tconvs
Tregs
Gated live CD45+
Surface CD74
Gated CD4+
Surface CD74
Surface CD74
NSCLC
24.8
CD4
CD8
27.8
0.31
89.2
10.2
2.74
9.68
TNBC
36.9
CD4
CD8
24.8
0.27
82.0
17.6
0.40
5.56
CD4-BV786
CD74-FITC
CD3-BV650
CD3-BV650
Foxp3-PEcf593
CD3-BV650
D
FACS: NSCLC patient
Blood
TDLN
Tumor
CD8+
1.66
3.59
1.64
Tconvs
0.69
0.75
3.44
Tregs
6.11
3.03
8.68
CD74-FITC
CD4-BV786
CD74+ cells (%)
Among Live CD3+ cells
Blood
TDLN
Tumor
Tissues from NSCLC patient
CD8+ cells
CD4+Foxp3- cells
CD4+Foxp3- cells
E
FACS: NSG mouse engrafted with MDA-MB231
Spleen
Tumor
CD8+
10,8
13,8
Tconvs
11,6
37,7
Tregs
14,8
51,9
CD74-APCcy7
CD4-BV786
CD74+ cells (%)
Among TCRβ+ cells
Spleen
Tumor
CD8+ cells
CD4+Foxp3- cells
CD4+Foxp3+ cells
F
FACS: in vitro stimulation
CD74+ cells (%)
Among CD4+Foxp3+ cells
Level in tumTregs
Untreated
IFNα
IFNγ
HCC827
MCF-7
MDA-MB231
derived tumor-supernatant

Figure. S1: CD74 is overexpressed in Tregs from different tumor types compared to Tconvs and CD8+ cells.

A: Single-cell RNA sequencing (scRNAseq) of CD4+ T cells from blood of 5 non-small cell lung cancer (NSCLC) patients (from Tosello, Richer et al., under review, accession code EGAS50000000293). Volcano plot (P-value versus fold change) of the gene expression profile of Tregs and Tconvs. Genes highlighted in blue are overexpressed by peripheral Tregs and highlighted in green by peripheral Tconvs. **B**: Bulk RNA sequencing (bulk-RNAseq) of CD4+ T cells from tumor (5 colorectal carcinoma (CRC) patients from De Simone et al. dataset[48] (GEO access: GSE50760), and 2 luminal- breast cancer (TNBC) patients from Nunez, Tosello et al. dataset (ArrayExpress access: E-MTAB-9112, *Núñez, N. G. et al. Tumor invasion in draining lymph nodes is associated with Treg accumulation in breast cancer patients. Nat Commun* ***11****, 3272 (2020))*. Violin plot of CD74 normalized expression in Tregs versus Tconvs from tumor samples of CRC patients (left) and TNBC patients (right). **C**: Representative dotplots to illustrate the gating strategy used to distinguish T-cell subsets from total cells. **D**: Representative dotplots (top) and quantification (bottom) of CD74-surface expression among CD8+ cells (red), CD4+ Tconvs (green) and Tregs (blue) from blood, tumor-draining lymph node and tumor of paired NSCLC patients. **E**: NSG mice (n=5) were engrafted with 5x106 MDAMB231 cells, and 10 days later, expanded Tregs and fresh PBMCs were co-transferred intravenously in tumor-bearing mice. Sixteen days after tumor-cell injection, the spleens and tumors were analyzed by FACS. Representative dotplots (left) and quantification (right) of CD74-surface expression among CD8+ cells (red), CD4+ Tconvs (green) and Tregs (blue) from spleens and tumors of MDA-MB231 tumor-bearing NSG mice. **F**: Quantification of CD74 surface expression on expanded Tregs untreated or stimulated during 72h with IFN**α** (1000IU/mL, n=5), IFN**γ** (100ng/mL, n=4) or with tumor-derived supernatant from the following cell-lines: HCC827 (NSCLC, n=4), MCF-7 (Breast, n=4) or MDA-MB231 (TNBC, n=4). Statistical analyses are performed using an unpaired t-test; with pVal < **:0,01; ***:0,001 (**B**) and horizontal lines represent median (**B** and **E-F**). Source data are provided as Source Data file.

Figure.S2

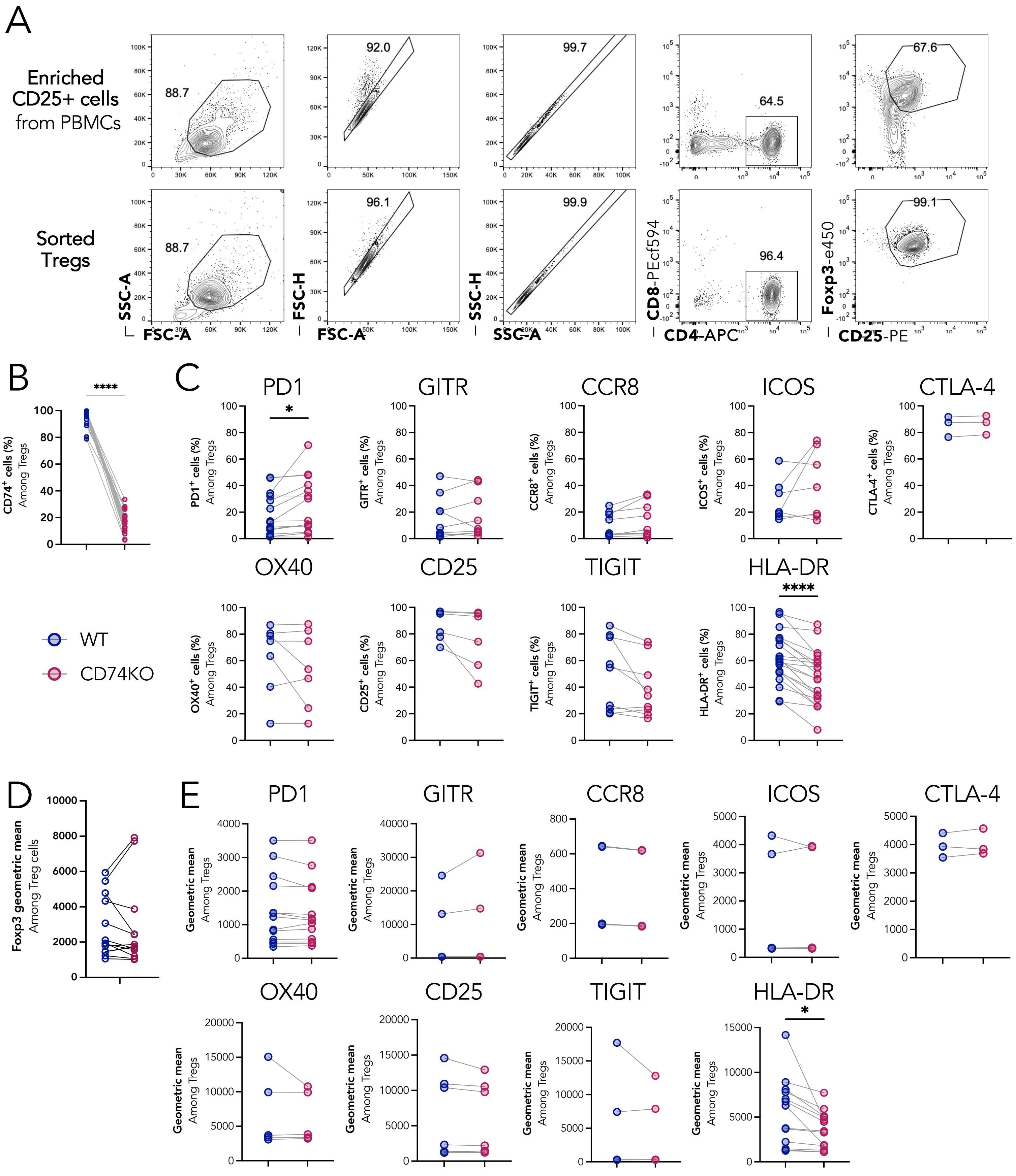

A
Enriched CD25+ cells from PBMCs
Sorted Tregs
88.7
92.0
99.7
64.5
67.6
88.7
96.1
99.9
96.4
99.1
SSC-A
FSC-A
FSC-H
FSC-A
SSC-H
SSC-A
CD8-PEcf594
CD4-APC
Foxp3-e450
CD25-PE
B
****
CD74+ cells (%) Among Tregs
C
PD1
GITR
CCR8
ICOS
CTLA-4
*
PD1+ cells (%) Among Tregs
GITR+ cells (%) Among Tregs
CCR8+ cells (%) Among Tregs
ICOS+ cells (%) Among Tregs
CTLA-4+ cells (%) Among Tregs
OX40
CD25
TIGIT
HLA-DR
****
OX40+ cells (%) Among Tregs
CD25+ cells (%) Among Tregs
TIGIT+ cells (%) Among Tregs
HLA-DR+ cells (%) Among Tregs
WT
CD74KO
D
Foxp3 geometric mean Among Treg cells
E
PD1
GITR
CCR8
ICOS
CTLA-4
Geometric mean Among Tregs
OX40
CD25
TIGIT
HLA-DR
*

Figure. S2 : Phenotypic characterization of WT and CD74KO Tregs.

A: Representative dotplots to illustrate the purity of Tregs in CD25 enriched cells (top) or in sorted CD4+CD127-CD25hi cells (bottom). **B**: Quantification of CD74 intracellular expression 5 days after electroporation with CRISPR-Cas9 RNPs targeting CD74 exons (n=23). **C**: Quantification of the percentage of positive cells among in WT (blue) or CD74KO (red) Tregs for PD1 (n=14), GITR (n=9), CCR8 (n=9), ICOS (n=7), CTLA-4 (n=3), OX40 (n=7), CD25 (n=7), TIGIT (n=9) and HLA-DR (n=20). **D**: Quantification of Foxp3 geometric mean in WT (blue) or CD74KO (red) Tregs (n=13). **E**: Quantification of the geometric mean of the indicated marker in the positive population for this marker among CD74KO vs WT Tregs (PD1 (n=13), GITR (n=5), CCR8 (n=5), ICOS (n=5), CTLA-4 (n=3), OX40 (n=5), CD25 (n=7), TIGIT (n=5) and HLA-DR (n=13)) Statistical analyses are performed using a paired t-test; with pVal < *:0,1; ***:0,001; ****:0,0001. Source data are provided as Source Data file.

Figure.S3

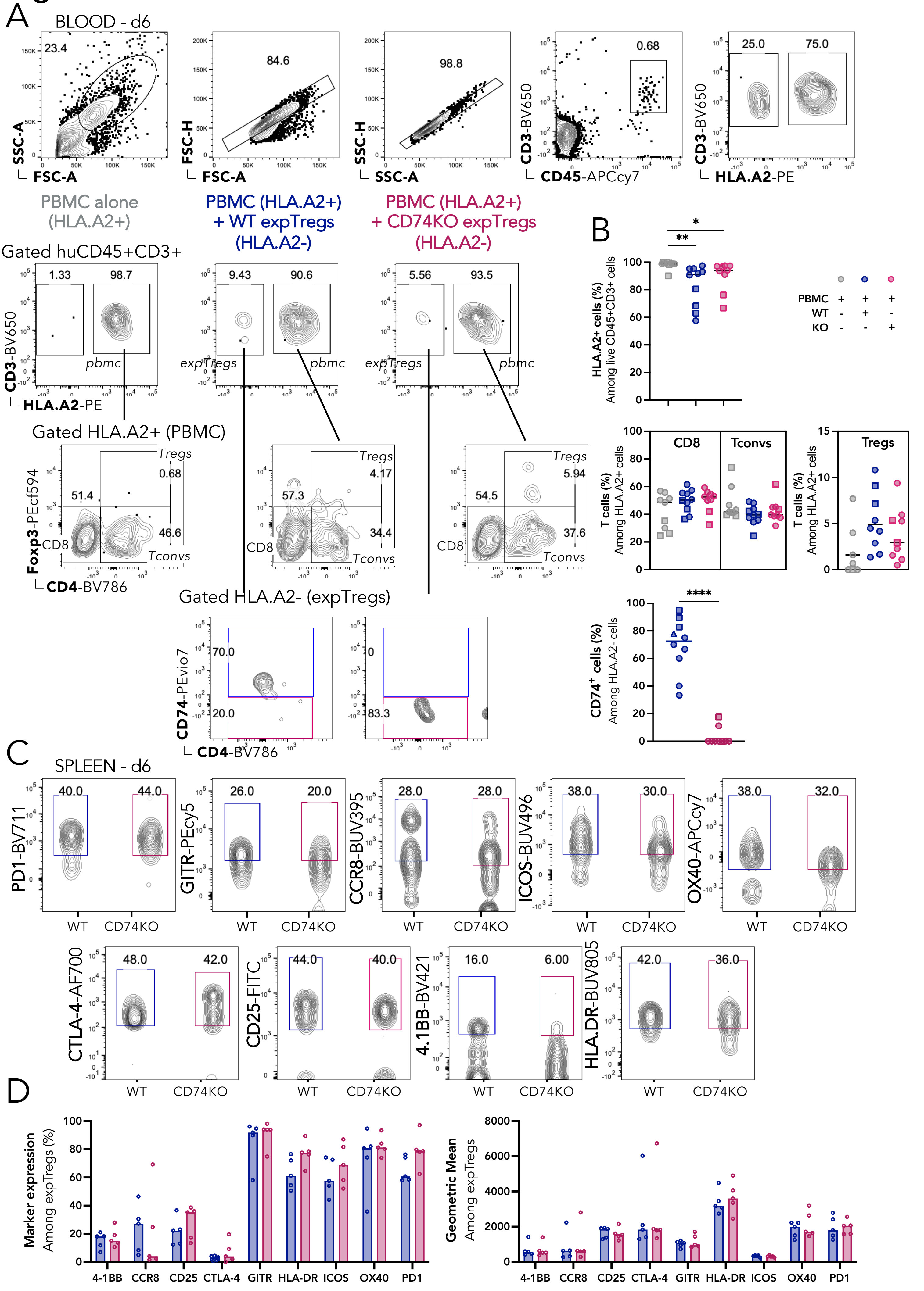
A
BLOOD - d6
SSC-A
FSC-A
FSC-H
SSC-H
CD3-BV650
CD45-APCcy7
HLA.A2-PE
23.4
84.6
98.8
0.68
25.0
75.0
PBMC alone (HLA.A2+)
PBMC (HLA.A2+) + WT expTregs (HLA.A2-)
PBMC (HLA.A2+) + CD74KO expTregs (HLA.A2-)
Gated huCD45+CD3+
1.33
98.7
9.43
90.6
5.56
93.5
pbmc
expTregs
Gated HLA.A2+ (PBMC)
Foxp3-PEcf594
CD4-BV786
Tregs
Tconvs
CD8
0.68
51.4
46.6
4.17
57.3
34.4
5.94
54.5
37.6
Gated HLA.A2- (expTregs)
CD74-PEvio7
70.0
20.0
0
83.3
B
HLA.A2+ cells (%)
Among live CD45+CD3+ cells
PBMC + + +
WT - + -
KO - - +
T cells (%)
Among HLA.A2+ cells
CD8
Tconvs
Tregs
CD74+ cells (%)
Among HLA.A2- cells
C
SPLEEN - d6
PD1-BV711
40.0
44.0
GITR-PEcy5
26.0
20.0
CCR8-BUV395
28.0
28.0
ICOS-BUV496
38.0
30.0
OX40-APCcy7
38.0
32.0
CTLA-4-AF700
48.0
42.0
CD25-FITC
44.0
40.0
4.1BB-BV421
16.0
6.00
HLA.DR-BUV805
42.0
36.0
WT
CD74KO
D
Marker expression
Among expTregs (%)
Geometric Mean
Among expTregs
4-1BB
CCR8
CD25
CTLA-4
GITR
HLA-DR
ICOS
OX40
PD1

Figure. S3 : Description of T cells in NSG mice transferred with PBMCs alone or together with WT or CD74KO expTregs.

A: Representative dotplots to illustrate the gating strategy to identify HLA.A2+ PBMCs from HLA.A2- expTregs. **B**: Quantification of T cell proportions 6 days after injection, in the spleen of mice receiving PBMCs alone (gray), together with WT expTregs (blue), or with CD74KO expTregs (red) (2 donors, o n=9 or ☐ n=10). **C**: Representative dotplots of Treg-markers' expression in spleen with concatenated WT and CD74KO expTregs from the same donor (concatenation on the similar number of pure Tregs, WT and CD74KO Tregs represent each 50%). **D**: Quantification of the percentage of cells expressing (left) and the geometric mean (right) of the marker expression in WT (n=5) and CD74KO (n=5) expTregs. Statistical analyses are performed using a paired t-test; with pVal < *:0,1; **:0,01; ****:0,0001 (**B** and **D**). Source data are provided as a Source Data file.

Figure.S4

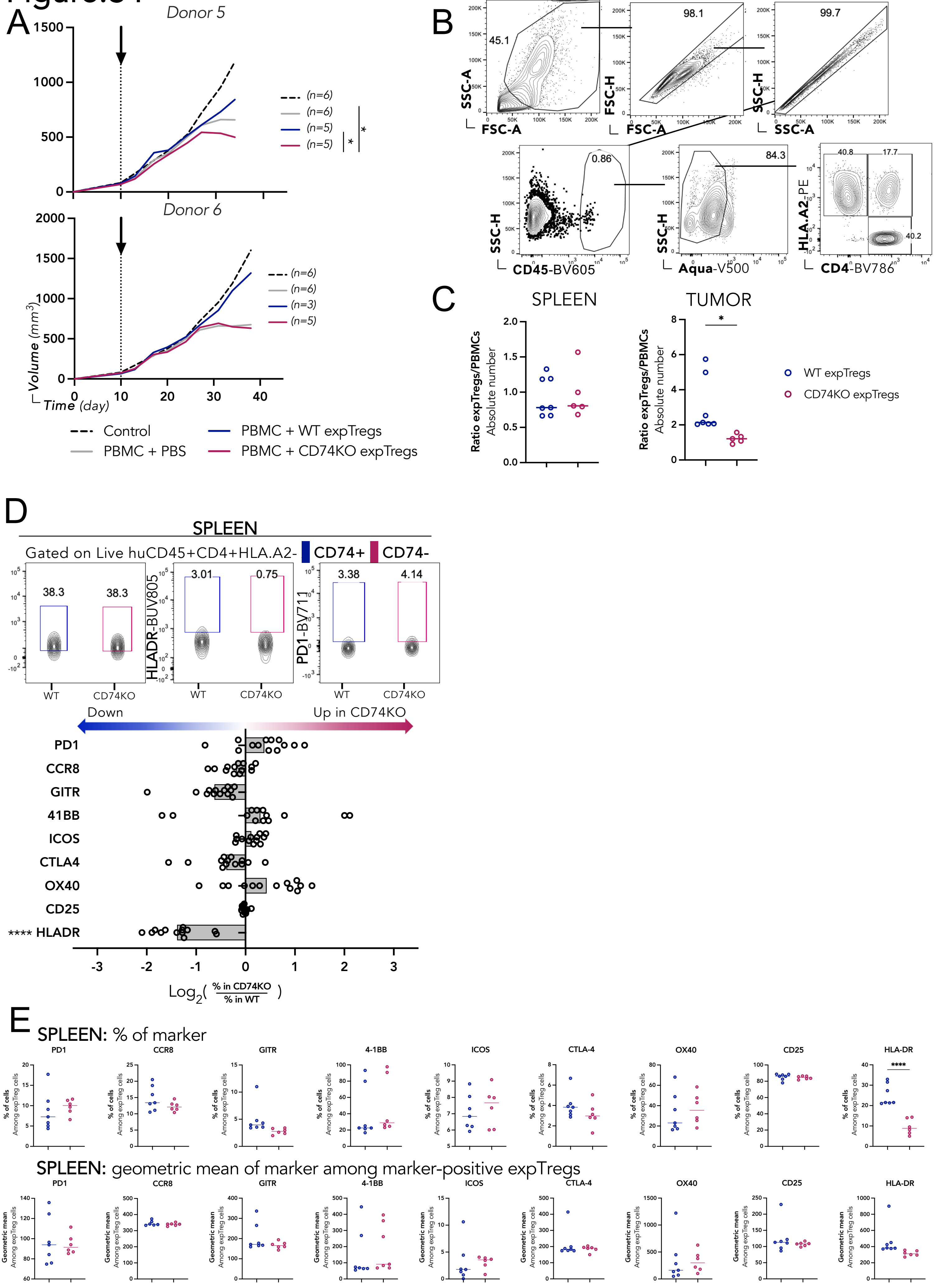
A
Donor 5
Donor 6
(n=6)
(n=6)
(n=5)
(n=5)
(n=3)
*
Volume (mm3)
Time (day)
Control
PBMC + PBS
PBMC + WT expTregs
PBMC + CD74KO expTregs
B
SSC-A
FSC-A
FSC-H
SSC-H
45.1
98.1
99.7
0.86
84.3
40.8
17.7
40.2
CD45-BV605
Aqua-V500
HLA.A2-PE
CD4-BV786
C
SPLEEN
TUMOR
Ratio expTregs/PBMCs
Absolute number
WT expTregs
CD74KO expTregs
D
SPLEEN
Gated on Live huCD45+CD4+HLA.A2-
CD74+
CD74-
38.3
38.3
3.01
0.75
3.38
4.14
HLADR-BUV805
PD1-BV711
WT
CD74KO
Down
Up in CD74KO
PD1
CCR8
GITR
41BB
ICOS
CTLA4
OX40
CD25
**** HLADR
Log2(% in CD74KO / % in WT)
E
SPLEEN: % of marker
PD1
CCR8
GITR
4-1BB
ICOS
CTLA-4
OX40
CD25
HLA-DR
****
% of cells Among expTreg cells
SPLEEN: geometric mean of marker among marker-positive expTregs
Geometric mean Among expTreg cells

Figure.S4-following

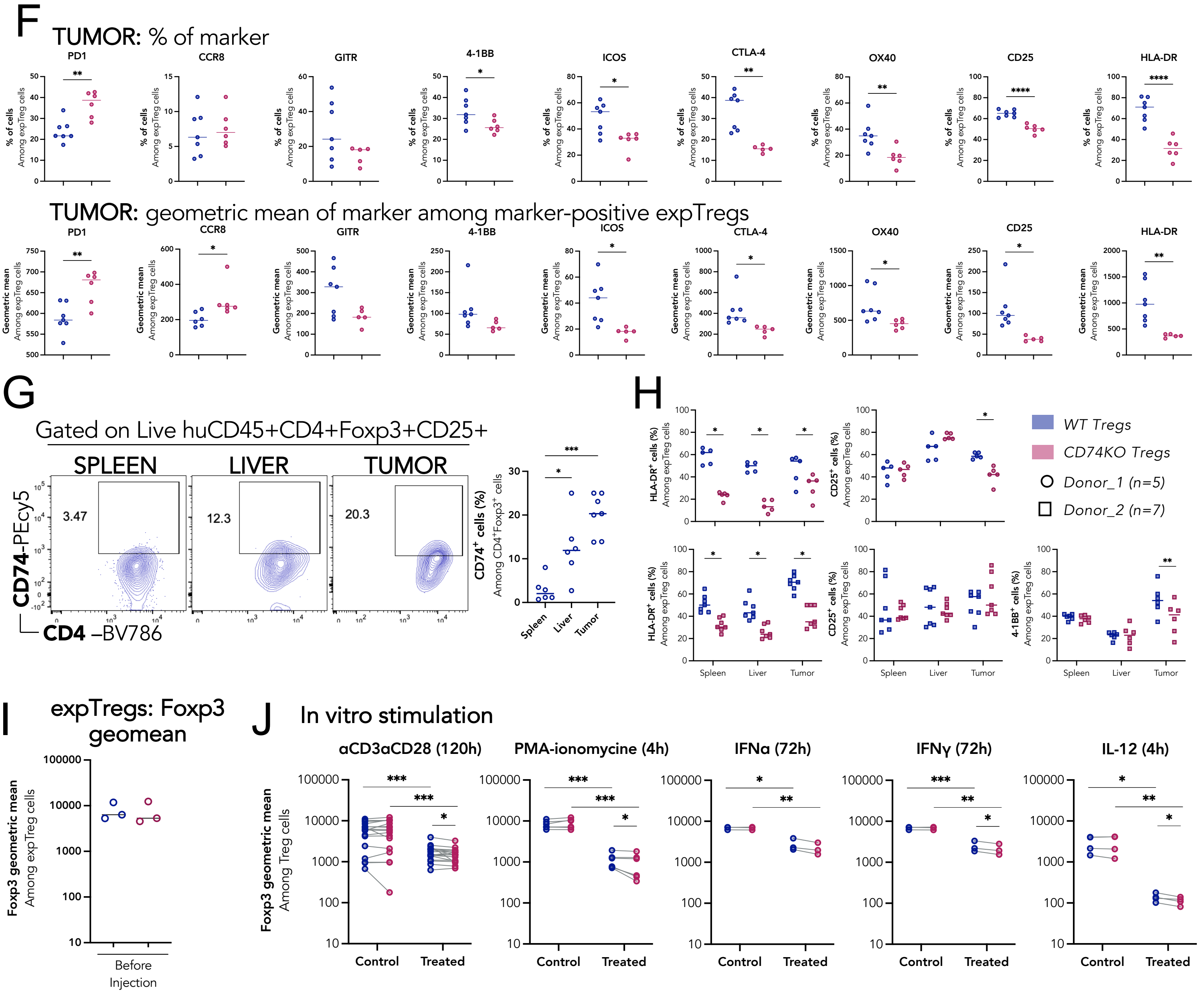

Figure. S4 : Repartition of T cells in tumor-bearing mice transferred with PBMCs alone or together with WT expTregs or CD74KO expTregs.

MDA-MB231 tumor cells are engrafted subcutaneously in the flank of immunodeficient NSG mice. 10 days later, HLA.A2+ PBMCs are injected intravenously alone (gray) or together with WT (blue), or CD74KO (red) expanded and then frozen HLA.A2- Tregs, a group of mice is not injected as a control (black). **A**: Median of tumor-growth curves of each group with 2 distinct donors. **B**: Representative dotplots to illustrate the gating strategy to identify HLA.A2+ PBMCs from HLA.A2- expTregs in spleen. **C**: Ratio of absolute numbers of expTregs (HLA.A2-)/PBMCs (HLA.A2+) in the spleen and tumors 6 days after cell injection (WT expTregs in blue, n=7 and CD74KO expTregs in red, n=5). **D**: Representative dotplots of CD25, HLA-DR and PD1 surface expression in concatenated samples of WT and CD74KO splenic expTregs (top) and quantification of the markers in expTregs, represented as the ratio among CD74KO versus WT expTregs (bottom) (n=13). **E-F**: Quantification of the percentage (top) or the geometric mean level (bottom) of the indicated marker among CD74KO vs WT expTregs from spleens (**E**) and tumors (**F**) (n=6-7). **G**: Representative FACS dot plots (left) and quantification (right) of % of cells expressing CD74 at the surface of CD4+ Foxp3+ Tregs in spleens, livers and tumors from MDA-MB231 tumor-bearing NSG mice. **H**: Quantification of the percentage of the indicated marker among CD74KO vs WT expTregs from spleens, livers, and tumors (2 donors, o n=5 or □ n=7). **I**: Quantification of Foxp3 geometric mean in WT and CD74KO expanded Tregs before injection (n=3). **J**: Quantification of Foxp3 geometric mean in WT and CD74KO Tregs after stimulation with **α**CD3**α**CD28 (120h, 1bead:1cell, n=17), PMA-ionomycine (4h, 100ng/mL-1µg/mL, n=6), IFN**α** (72h, 1000IU/mL, n=3), IFN**γ** (72h, 100ng/mL, n=3) or IL-12 (4h, 20ng/mL, n=4). Statistical analyses are performed using an unpaired t-test (**A-C** and **E-H**); or paired t-test (**D-J**); with pVal < *:0,1; **:0,01; ***:0,001; ****:0,0001. Source data are provided as a Source Data file.

Figure.S5

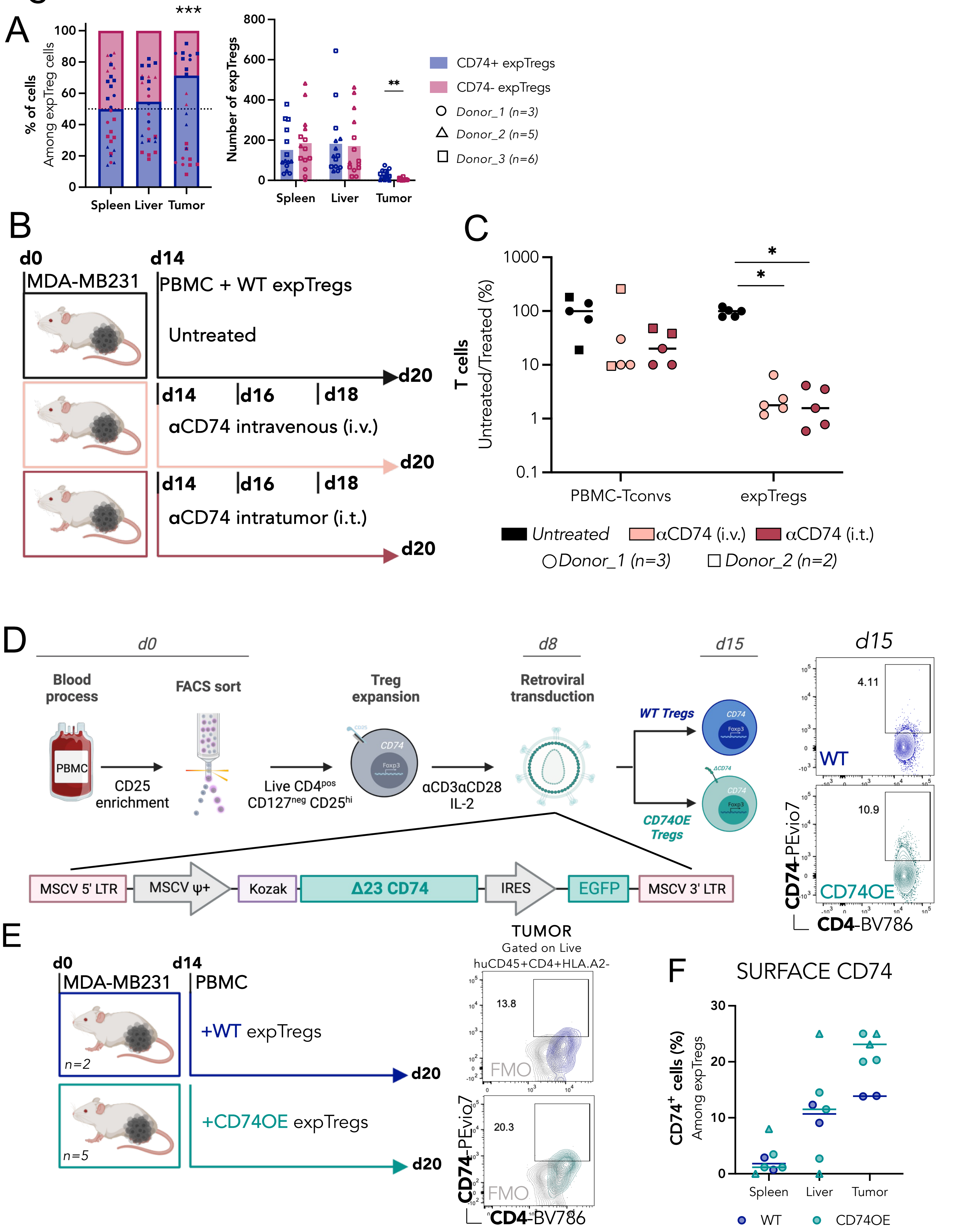

A
***
% of cells
Among expTreg cells
Spleen Liver Tumor
Number of expTregs
**
Spleen
Liver
Tumor
CD74+ expTregs
CD74- expTregs
Donor_1 (n=3)
Donor_2 (n=5)
Donor_3 (n=6)
B
d0
MDA-MB231
d14
PBMC + WT expTregs
Untreated
d20
d14 d16 d18
αCD74 intravenous (i.v.)
d20
d14 d16 d18
αCD74 intratumor (i.t.)
d20
C
T cells
Untreated/Treated (%)
*
*
PBMC-Tconvs
expTregs
Untreated
αCD74 (i.v.)
αCD74 (i.t.)
Donor_1 (n=3)
Donor_2 (n=2)
D
d0
d8
d15
Blood process
FACS sort
Treg expansion
Retroviral transduction
PBMC
CD25 enrichment
Live CD4pos CD127neg CD25hi
αCD3αCD28 IL-2
WT Tregs
CD74OE Tregs
MSCV 5' LTR
MSCV ψ+
Kozak
Δ23 CD74
IRES
EGFP
MSCV 3' LTR
d15
4.11
WT
10.9
CD74OE
CD74-PEvio7
CD4-BV786
E
d0
MDA-MB231
d14
PBMC
+WT expTregs
n=2
d20
+CD74OE expTregs
n=5
d20
TUMOR
Gated on Live
huCD45+CD4+HLA.A2-
13.8
FMO
20.3
FMO
CD74-PEvio7
CD4-BV786
F
SURFACE CD74
CD74+ cells (%)
Among expTregs
Spleen
Liver
Tumor
WT
CD74OE

Figure.S5-following

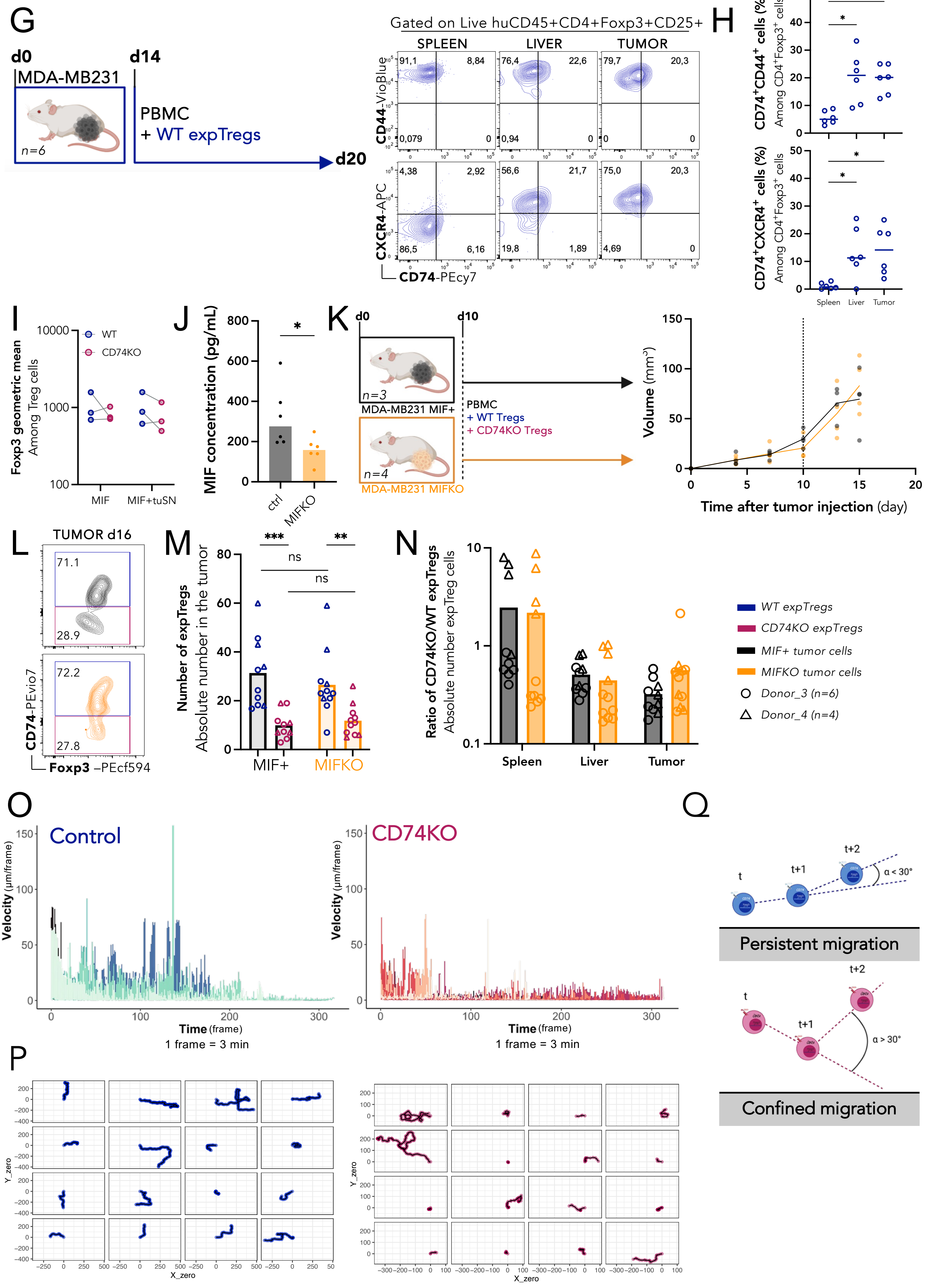

Figure. S5 : Tumor invasion, MIF independency, velocity and trajectory of WT and CD74KO Tregs.

A: Quantification of WT and CD74KO expTregs in tissue of tumor-bearing mice co-injected with HLA.A2+ PBMCs, HLA.A2- WT and -CD74KO expTregs (3 donors, ○ n=3, △ n=5 or □ n=6). **B-C**: Tumor-bearing NSG injected with HLA.A2+ PBMCs and HLA.A2- WT expTregs were treated with anti-CD74 (aCD74) i.v. or i.t. **C**: Tumor-infiltrating PBMC-derived Tconvs and expTregs, calculated as ratio of absolute number of tumor-infiltrating CD4+ T-cells in untreated versus anti-CD74-treated mice (2 donors, ○ n=3 or □ n=2). **D**: FACS-sorted Tregs were expanded for 8 days and transduced with a retrovirus containing GFP (WT) or GFP plus with a 23-aminoacid truncated CD74 mutant to overexpress CD74 at the surface (CD74OE). Representative dot-plots of CD74 surface expression 7 days after transduction. **E-F**: Tumor-bearing NSG received HLA.A2+ PBMCs with WT (blue) or CD74OE (green) expanded-HLA.A2- Tregs and analyzed by FACS. Representative dotplots of surface CD74 in expTregs (**E**) and quantification (**F**, 2 donors, ○ n=3 or △ n=2). **G-H**: Tumor- bearing NSG mice received PBMCs with expTregs and tissues were analyzed by FACS for CD44 and CXCR4 expression in Tregs. Representative FACS dotplots (**G**) and percentages of CD44 (top) and CXCR4 (bottom) co-expression with CD74 at the surface of expTregs (**H**, n=6). **I**: Quantification of Foxp3 geometric mean in WT and CD74KO Tregs after in vitro stimulation with MDA-MB231-tumor cell derived supernatant (tuSN) with or without soluble MIF (48h, 200ng/mL) (n=3). **J**: Concentration of MIF in supernatant of control or MIF-KO MDA-MB231 cells 4 days after CRISPRCas9 deletion. **K-N**: : MIFKO (n=3) or control (MIF+, n=4) MDA-MB231 tumor-bearing NSG were injected with PBMCs, WT expTregs and CD74KO expTregs. Tumor size of MIFKO or ctrl tumors (**K**). Representative dotplots (**L**), quantification of absolute number of WT (blue) and CD74KO (red) expTregs among total expTregs in the MIFKO (orange) or MIF+ ctrl (black) tumor (**M**), and ratio of absolute number of CD74KO expTregs versus WT expTregs in tissues (**N**). **M-N**: 2 donors, ○ n=6 or △ n=4. **O-P**: WT and CD74KO Tregs from 3 donors are expanded and activated for 14 days before loading into microchannel or micropillar gels. **O**: Speed according to time of WT and CD74KO Tregs loaded in microchannel gels. **P**: Individual trajectory of the top16 longest paths of WT (blue) and CD74KO (red) Tregs loaded in micropillar gel. **Q**: Schematic representation defining confined versus persistent migration in the 2-dimension migratory model. Statistical analyses are performed using an unpaired t-test with pVal < *:0,1; **:0,01; ***:0,001. Source data are provided as a Source Data file. Figures S5D, E, G, K were created with BioRender.com.

Figure.S6

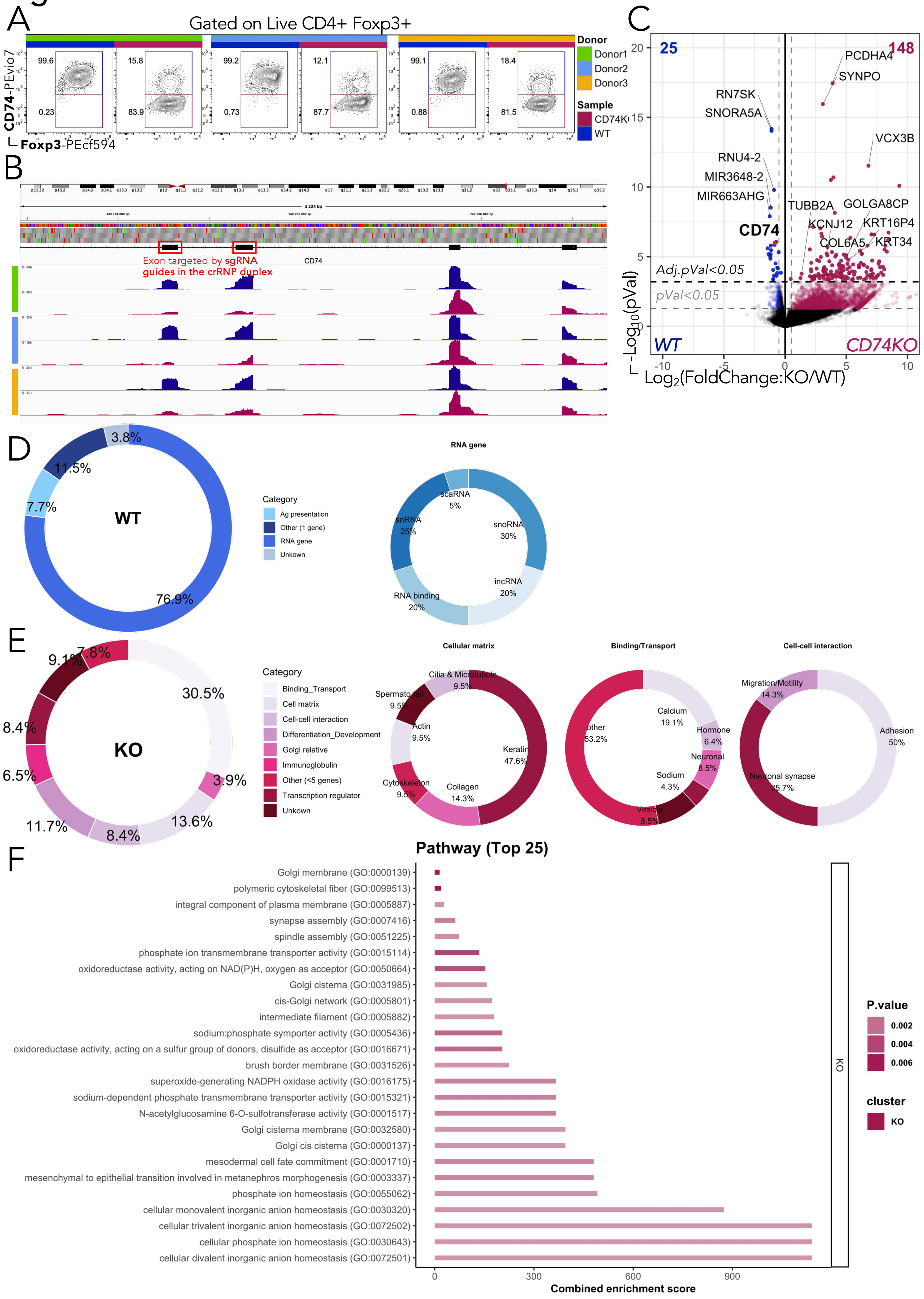
A
Gated on Live CD4+ Foxp3+
CD74-PEvio7
Foxp3-PEcf594
99.6
0.23
15.8
83.9
99.2
0.73
12.1
87.7
99.1
0.88
18.4
81.5
Donor
Donor1
Donor2
Donor3
Sample
CD74KO
WT
B
Exon targeted by sgRNA guides in the crRNP duplex
CD74
C
25
148
PCDHA4
SYNPO
RN7SK
SNORA5A
VCX3B
RNU4-2
MIR3648-2
MIR663AHG
TUBB2A
GOLGA8CP
KCNJ12
KRT16P4
CD74
COL6A5
KRT34
Adj.pVal<0.05
pVal<0.05
WT
CD74KO
-Log10(pVal)
Log2(FoldChange:KO/WT)
D
WT
3.8%
11.5%
7.7%
76.9%
Category
Ag presentation
Other (1 gene)
RNA gene
Unkown
RNA gene
scaRNA 5%
snoRNA 30%
snRNA 25%
incRNA 20%
RNA binding 20%
E
KO
9.1%
7.8%
30.5%
8.4%
6.5%
3.9%
11.7%
8.4%
13.6%
Category
Binding_Transport
Cell matrix
Cell-cell interaction
Differentiation_Development
Golgi relative
Immunoglobulin
Other (<5 genes)
Transcription regulator
Unkown
Cellular matrix
Cilia & Microtubule 9.5%
Spermato tail 9.5%
Actin 9.5%
Keratin 47.6%
Cytoskeleton 9.5%
Collagen 14.3%
Binding/Transport
Calcium 19.1%
other 53.2%
Hormone 6.4%
Neuronal 8.5%
Sodium 4.3%
Vesicle 8.5%
Cell-cell interaction
Migration/Motility 14.3%
Adhesion 50%
Neuronal synapse 35.7%
F
Pathway (Top 25)
Golgi membrane (GO:0000139)
polymeric cytoskeletal fiber (GO:0099513)
integral component of plasma membrane (GO:0005887)
synapse assembly (GO:0007416)
spindle assembly (GO:0051225)
phosphate ion transmembrane transporter activity (GO:0015114)
oxidoreductase activity, acting on NAD(P)H, oxygen as acceptor (GO:0050664)
Golgi cisterna (GO:0031985)
cis-Golgi network (GO:0005801)
intermediate filament (GO:0005882)
sodium:phosphate symporter activity (GO:0005436)
oxidoreductase activity, acting on a sulfur group of donors, disulfide as acceptor (GO:0016671)
brush border membrane (GO:0031526)
superoxide-generating NADPH oxidase activity (GO:0016175)
sodium-dependent phosphate transmembrane transporter activity (GO:0015321)
N-acetylglucosamine 6-O-sulfotransferase activity (GO:0001517)
Golgi cisterna membrane (GO:0032580)
Golgi cis cisterna (GO:0000137)
mesodermal cell fate commitment (GO:0001710)
mesenchymal to epithelial transition involved in metanephros morphogenesis (GO:0003337)
phosphate ion homeostasis (GO:0055062)
cellular monovalent inorganic anion homeostasis (GO:0030320)
cellular trivalent inorganic anion homeostasis (GO:0072502)
cellular phosphate ion homeostasis (GO:0030643)
cellular divalent inorganic anion homeostasis (GO:0072501)
KO
0
300
600
900
Combined enrichment score
P.value
0.002
0.004
0.006
cluster
KO

Figure.S6-following

G

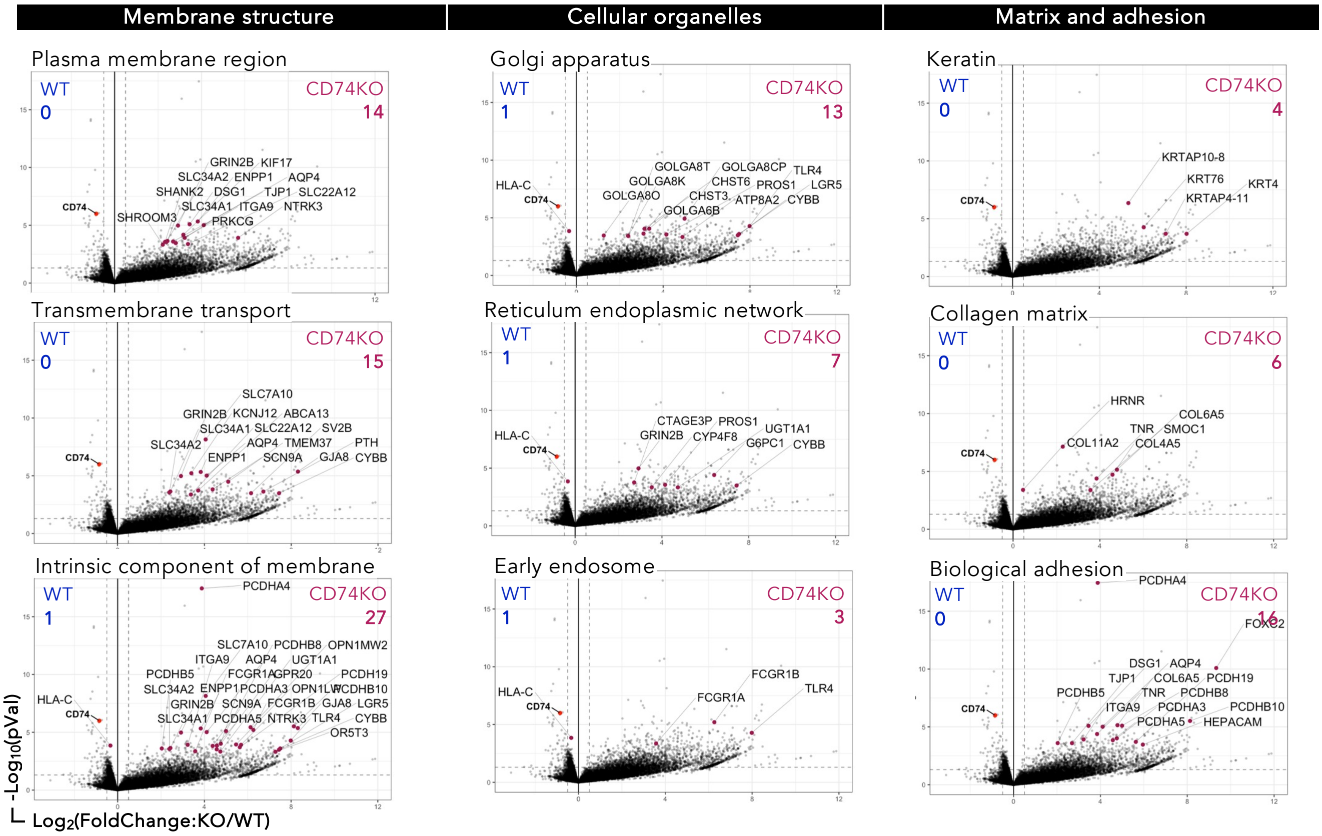

Figure. S6 : Transcriptomic analysis of CD74KO and WT Tregs.

FACS sorted Tregs from 3 donors are expanded for 7 days and electroporated with two CRISPR-Cas9 RNPs targeting CD74 (CD74KO) or one control RNP (WT). Electroporated cells are expanded for 14 days and the RNA from WT and CD74KO Tregs are harvested to perform a bulk-RNA sequencing. **A**: Dotplots depicting CD74 expression among the Tregs (WT and CD74KO) used for RNA sequencing. **B**: Visualization of reads aligned on CD74 locus from Hg38 reference genome. **C**: Volcano plot (Pvalue versus fold change) showing the differential gene expression between CD74KO and WT Tregs. On y-axis, the dashed lines represent adjusted P value at 0.05 (black) and Pvalue at 0.05 (gray). Upregulated genes relative to WT Tregs (downregulated by CD74KO) are colored in blue and upregulated genes relative to CD74KO Tregs are colored in red, number of differentially expressed genes (DEGs) are annotated in the respective color. **D-E**: Proportion of the genes associated to biological categories based on the known biology of DEGs in WT Tregs (**D**) or in CD74KO Tregs (**E**). F: Top25 pathways of the EnrichR analysis based on the 148 DEGs upregulated by CD74KO Tregs. **G**: Volcano plots highlighting the genes associated to the pathways indicated in the title from the GSEA library. Source data are provided as a Source Data file.

Figure.S7

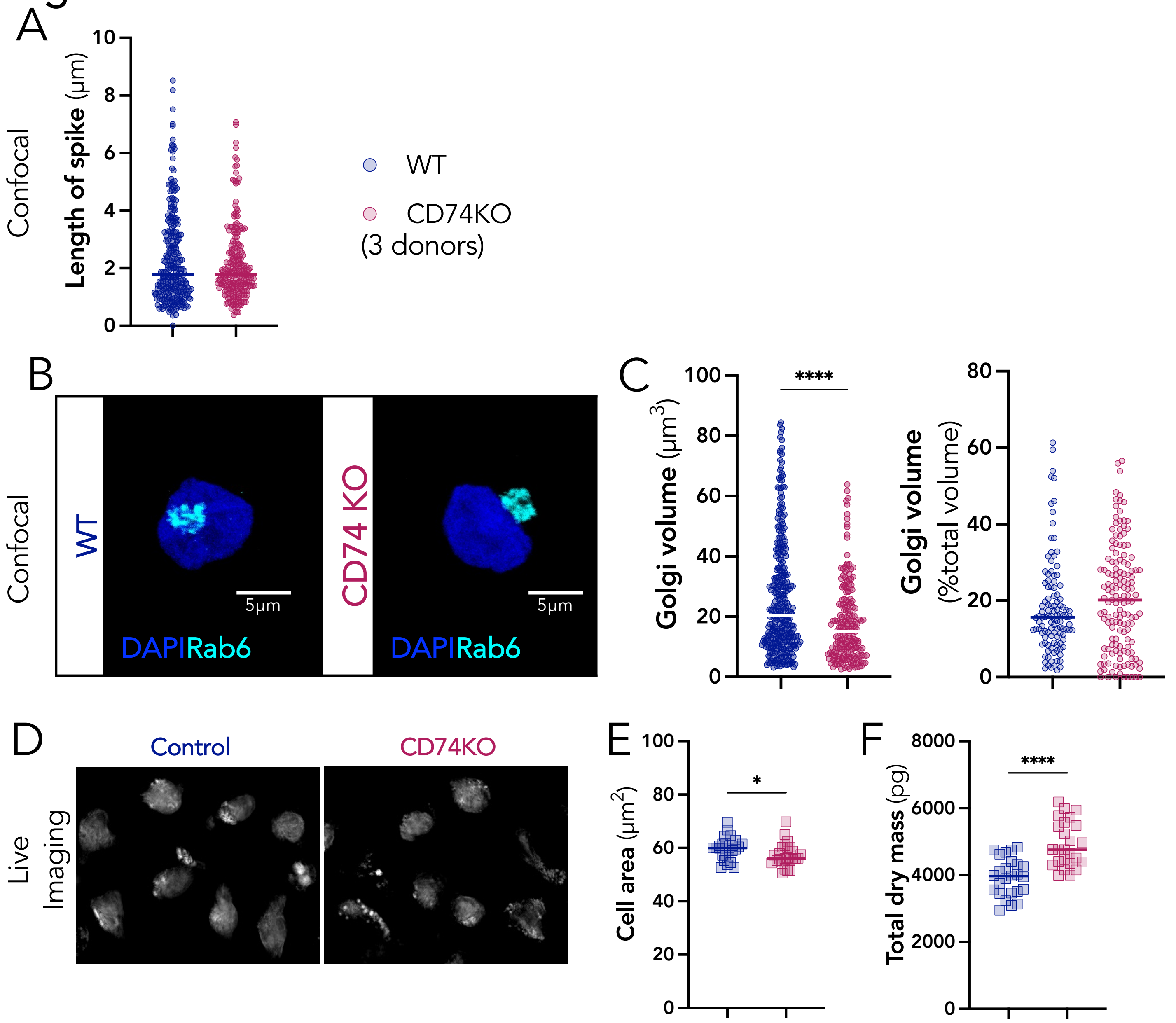

Figure. S7 : Perturbed cell shape, organelle organization and cell interaction of CD74KO Tregs.

A: Quantification of the length of the spikes surrounding Tregs based on phalloidin staining. **B**: Confocal images showing the localization of DAPI (blue) and Rab6 (cyan) in a z-stack projection of WT and CD74KO Tregs. **C**: Measure of the total volume of Golgi apparatus (left) and of the percentage of volume occupied by Golgi apparatus in cells (right), analyzed with the Rab6 staining for Golgi apparatus volume and the phalloidin staining for cell volume. **D-F**: WT and CD74KO Tregs from 3 donors are expanded and activated for 14 days before being recorded by holotomography device for 24 hours in a 37°C-5%O_2 incubator. Data are recorded and analyzed by nanolive software. **D**: Snapshot of the live imaging acquired by holotomography of WT and CD74KO Tregs. **E-F**: Quantification from the holotomography imaging of the cell area (**E**) and the total dry mass (**F**) of WT and CD74KO Tregs. Statistical analyses are performed using an unpaired t-test; with pVal < *:0,1; **:0,01; ***:0,001; ****:0,0001 and horizontal lines represent median. **A-C**: Pooled data of the 3 donors. Source data are provided as a Source Data file.

Figure.S8

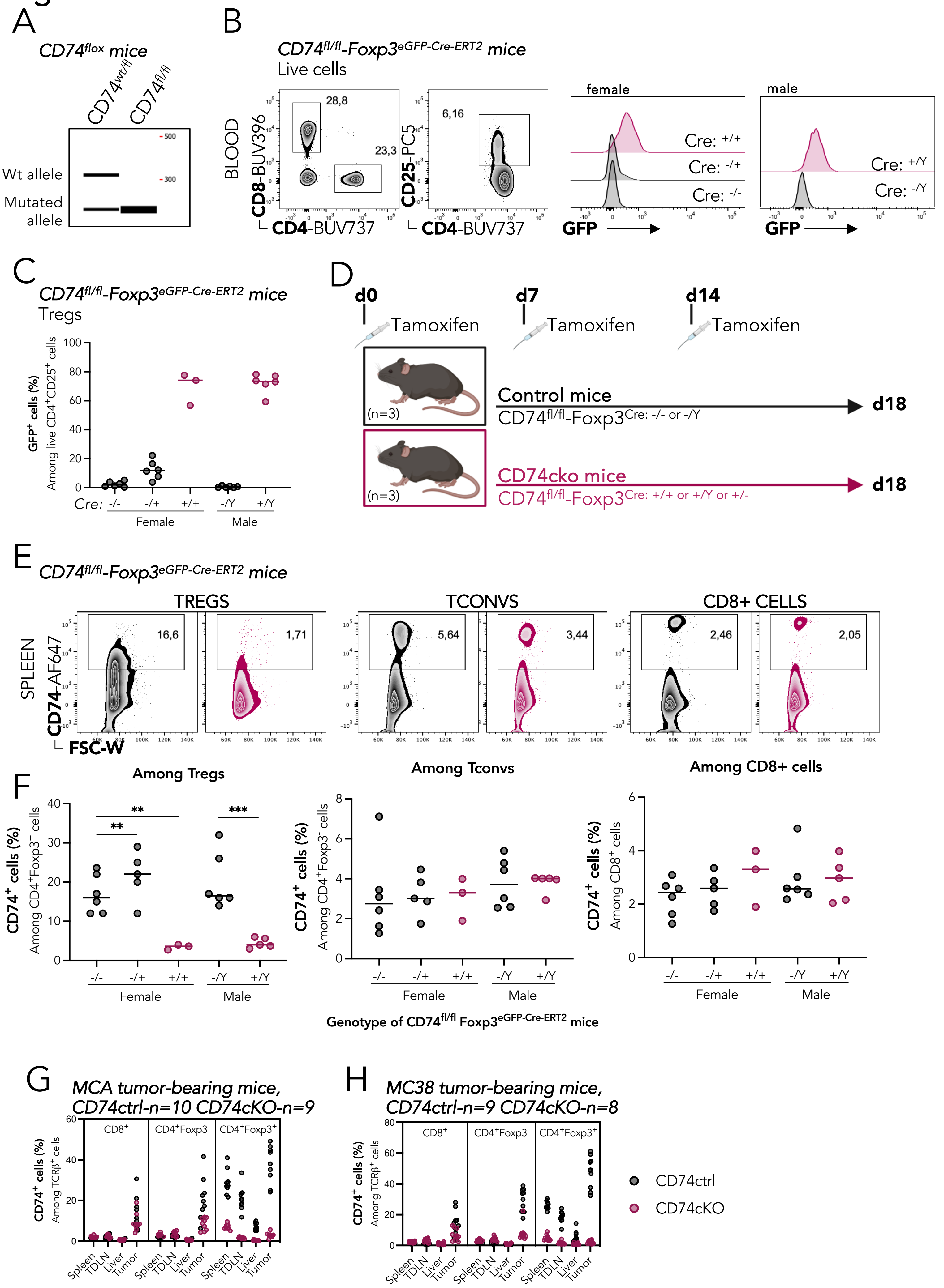
A
CD74flox mice
CD74wt/fl
CD74fl/fl
500
300
Wt allele
Mutated allele
B
CD74fl/fl-Foxp3eGFP-Cre-ERT2 mice
Live cells
BLOOD
CD8-BUV396
CD4-BUV737
28,8
23,3
CD25-PC5
6,16
female
male
Cre: +/+
Cre: -/+
Cre: -/-
Cre: +/Y
Cre: -/Y
GFP
C
CD74fl/fl-Foxp3eGFP-Cre-ERT2 mice
Tregs
GFP+ cells (%)
Among live CD4+CD25+ cells
Cre: -/- -/+ +/+ -/Y +/Y
Female
Male
D
d0
d7
d14
Tamoxifen
(n=3)
Control mice
CD74fl/fl-Foxp3Cre: -/- or -/Y
d18
CD74cko mice
CD74fl/fl-Foxp3Cre: +/+ or +/Y or +/-
E
CD74fl/fl-Foxp3eGFP-Cre-ERT2 mice
TREGS
TCONVS
CD8+ CELLS
SPLEEN
CD74-AF647
FSC-W
16,6
1,71
5,64
3,44
2,46
2,05
F
Among Tregs
Among Tconvs
Among CD8+ cells
CD74+ cells (%)
Among CD4+Foxp3+ cells
Among CD4+Foxp3- cells
Among CD8+ cells
**
**
***
-/- -/+ +/+ -/Y +/Y
Female
Male
Genotype of CD74fl/fl Foxp3eGFP-Cre-ERT2 mice
G
MCA tumor-bearing mice,
CD74ctrl-n=10 CD74cKO-n=9
H
MC38 tumor-bearing mice,
CD74ctrl-n=9 CD74cKO-n=8
CD8+
CD4+Foxp3-
CD4+Foxp3+
CD74+ cells (%)
Among TCRβ+ cells
Spleen
TDLN
Liver
Tumor
CD74ctrl
CD74cKO

Figure.S8: Generation and characterization of Foxp3-specific CD74 conditional knock-out mice (CD74cKO mice).

A: CD74flox mice were generated by the insertion of loxP sequences and PCR results illustrate the Wt versus mutant alleles in heterozygous (CD74wt/fl) or homozygous (CD74fl/fl) mice. **B-C**: CD74fl/fl mice were crossed with Foxp3eGFP-Cre-ERT2 mice. GFP expression was analyzed in CD4+CD25+ cells as markers for Treg in non-fixed blood cells from the resulting offsprings harboring the various possible genotypes for the GFP-Cre-ERT2 modification. **B**: Example of CD4+CD25+ gating strategy and GFP expression in Cre-ERT2-/- or -/+ or +/+ by flow cytometry. **C**: Quantification of % of GFP+ cells in CD4+CD25+ cells according to the mice genotypes (n=3 to 6 mice). **D-F**: Mice were treated by tamoxifen to induce the Cre-mediated deletion of CD74 and 4 days after the last injection its efficiency was analyzed in the spleen as the % of CD74+ (surface and intracellular) among CD4+Foxp3+ Treg cells, CD4+Foxp3- Tconv and CD8+ T cells. Representative dotplots (**E**) and quantification (**F**) of CD74 total expression among CD4+ Tregs (left), CD4+ Tconvs (middle), and CD8+ cells (right) according to the mice genotypes (n=3 to 6 mice). **G-H**: : CD74ctrl or CD74cKO male mice were treated with tamoxifen (on day 0, 7 and 14), grafted with murine tumor (MCA or MC38) on day 7 and tissues were analyzed on day 18 by flow cytometry. Quantification of CD74 total expression among CD8+ cells, Tconvs (CD4+Foxp3-) and Tregs (CD4+Fopx3+) in MCA-bearing mice (**G**) and in MC38-bearing mice (H). Statistical analyses are performed using an unpaired t-test (**F**) with pVal < *:0,1; **:0,01; ***:0,001; ****:0,0001 and horizontal lines represent median (**C** and **F**). Source data are provided as a Source Data file. Figure S8D was created with BioRender.com.

Table-S1. List of antibodies and markers used for cytometry and immunohistochemistry.

Figure	Marker	Fluorochrome	Staining step	Clone	Dilution	Provider	Catalog number
1 & S1	Live/Dead	Aqua	Surface		200	Invitrogen	L34957
	CD45	APCcy7	Surface	2D1	40	BD	557833
	CD3	BV650	Surface	OKT3	50	BioLegend	317324
	CD4	BV786	Surface	OKT4	150	Biolegend	317442
	Foxp3	PEcf594	Intracellular	236A/E7	25	BD	563955
	CD74	FITC	Surface	5-329	50	Invitrogen	11-0748-41
	CD74	PEvio770	Surface or Intracellular	5-329	50	Miltenyi	130-101-506
2 & S2	CTV		Cell tracer		1000	Invitrogen	C34557
	Live/Dead	Aqua	Surface		200	Invitrogen	L34957
	CD8	PEcf594	Surface	RPA-T8	800	BD	562282
	CD4	APC	Surface	M-T466	50	Miltenyi	130-113-250
	CD127	FITC	Surface	MB15-18C9	50	Miltenyi	130-113-409
	CD25	PE	Surface	M-A251	25	BD	555432
	Foxp3	PEcf594	Intracellular	236A/E7	25	BD	563955
	CD74	PEvio770	Intracellular	5-329	50	Miltenyi	130-101-506
	HLADR	BUV805	Surface	G46-6	100	BD	748338
	PD1	BV711	Surface	EH12.2H7	150	Biolegend	329928
	GITR	PEcy5	Surface	108-17	50	Biolegend	311608
	CCR8	BUV395	Surface	433H	25	BD	747573
	ICOS	BUV496	Surface	DX29	100	BD	750321
	OX40	APCVio770	Surface	REA621	50	Miltenyi	130-127-543
	CTLA4	APC	Intracellular	REA1003	50	Miltenyi	130-116-811
	TIGIT	PE	Surface	REA1004	25	Miltenyi	130-116-814
	41BB	BV421	Surface	4B4-1	50	BD	564091
3 & S3	Live/Dead	NIR	Surface		1000	Invitrogen	L10119
	CD4	BV786	Surface	OKT4	150	Biolegend	317442
	Foxp3	PEcf594	Intracellular	236A/E7	25	BD	563955
	CD25	FITC	Surface	M-A251	50	BD	555431
	HLADR	BUV805	Surface	G46-6	100	BD	748338
	PD1	BV711	Surface	EH12.2H7	150	Biolegend	329928
	CD74	PEvio770	Intracellular	5-329	50	Miltenyi	130-101-506
	GITR	PEcy5	Surface	108-17	50	Biolegend	311608
	CCR8	BUV395	Surface	433H	25	BD	747573

	ICOS	BUV496	Surface	DX29	100	BD	750321
	OX40	APCVio770	Surface	REA621	50	Miltenyi	130-127-543
	CTLA4	APC	Intracellular	REA1003	50	Miltenyi	130-116-811
	TIGIT	PE	Surface	REA1004	25	Miltenyi	130-116-814
	41BB	BV421	Surface	4B4-1	50	BD	564091
	HLAA2	PE	Surface	BB7.2	50	BD	558570
	CD45	BV605	Surface	HI30	100	Invitrogen	Q10051
	CD3	BV650	Surface	OKT3	50	BioLegend	317324
4 & S4	Live/Dead	NIR	Surface		1000	Invitrogen	L10119
	Live/Dead	Aqua	Surface		200	Invitrogen	L34957
	CD4	BV786	Surface	OKT4	150	Biolegend	317442
	Foxp3	PEcf594	Intracellular	236A/E7	25	BD	563955
	CD25	FITC	Surface	M-A251	50	BD	555431
	PD1	BV711	Surface	EH12.2H7	150	Biolegend	329928
	CD74	PEvio770	Intracellular	5-329	50	Miltenyi	130-101-506
	CD74	APCvio770	Intracellular	5-329	50	Miltenyi	130-101-534
	CD45	BV605	Surface	HI30	100	Invitrogen	Q10051
	HLAA2	PE	Surface	BB7.2	50	BD	558570
	CD4	BV786	Surface	OKT4	150	Biolegend	317442
	Foxp3	PEcf594	Intracellular	236A/E7	25	BD	563955
	HLADR	BUV805	Surface	G46-6	100	BD	748338
	PD1	BV711	Surface	EH12.2H7	150	Biolegend	329928
	GITR	PEcy5	Surface	108-17	50	Biolegend	311608
	CCR8	BUV395	Surface	433H	25	BD	747573
	ICOS	BUV496	Surface	DX29	100	BD	750321
	OX40	APCcy7	Surface	REA621	50	Miltenyi	130-127-543
	CTLA4	APC	Intracellular	REA1003	50	Miltenyi	130-116-811
	41BB	BV421	Surface	4B4-1	50	BD	564091
	IFNγ	V450	Intracellular	B27	50	BD	560371
	Caspase-3	PEcy7	Intracelluar	D3E9	50	cell signaling	64772S
5 & S5	CTV		Cell tracer		1000	Invitrogen	C34557
	CFSE		Cell tracer		5000	Invitrogen	C34554
	Live/Dead	NIR	Surface		200	Invitrogen	L10119
	CD4	BV786	Surface	OKT4	150	Biolegend	317442
	CD45	BV605	Surface	HI30	100	Invitrogen	Q10051
	Foxp3	PEcf594	Intracellular	236A/E7	25	BD	563955
	CD74	PEvio770	Surface	5-329	50	Miltenyi	130-101-506

	CD44	VioBlue	Surface	DB105	50	Miltenyi	130-113-899
	CXCR4	APC	Surface	12G5	25	Miltenyi	130-124-017
7 & S8	CD45.1	AF700	Surface	A20	200	BioLegend	110724
	CD45.2	APCcy7	Surface	104	150	BioLegend	109824
	TCRb	FITC	Surface	H57-597	400	BD	553171
	CD19	BV650	Surface	6D5	1600	BioLegend	115541
	CD4	BUV737	Surface	RM4-5	400	BD	564933
	CD8	BUV385	Surface	53-6.7	200	BD	563786
	CD25	PEcy5	Surface	PC61.5	800	eBioscience	15-0251-82
	Foxp3	PE	Intracellular	FJK-16s	200	eBioscience	12-5773-82
	CD74	AF647	Intracellular	In1/CD74	300	BioLegend	151004

**Figure**	**Marker**	**Fluorochrome**	**Clone**		**Provider**	**Catalog number**
**2, 6 & S7**	CD74	FITC	5-329	50	Invitrogen	11-0748-41
	HLA-DR	BV711	L243	100	Biolegend	307644
	Phalloidin	AF546	-	100	Thermo Fisher	A22283
	EEA1	-	CA5810	100	Cell Signalling	3288S
	Rab6	-	D37C7	100	Cell Signalling	9625S
	Anti-rabbit	AF568	polyclonal	200	Thermo Fisher	A11011
	Anti-rabbit	AF647	polyclonal	200	Thermo Fisher	A21245

Table-S2. Description of the different stimulation implement to challenge Tregs.

Stimulation	Final concentration	Time of activation	Provider	Catalog number
Phorbol 12-myristate 13-acetate (PMA)	100ng/mL	4h	Sigma Aldrich	P8139-1MG
Ionomycine calcium salt	1µg/mL	4h	Sigma Aldrich	I0634 - 1MG
Golgi Stop	1:1500	1h	BD bioscience	554724
Golgi Plug	1:1000	1h	BD bioscience	555029
Interferon-α	1000IU/mL	72h	Immunotools	11343594
Interferon-γ	100ng/mL	72h	Miltenyi	130-096-484
Interleukine-12	20ng/mL	4h	Immunotools	11349123
