# Supplementary material for: CD74 supports accumulation and function of regulatory T cells in tumors
Source: Nat Commun. 2024 May 3;15:3749. doi: 10.1038/s41467-024-47981-3 (PMC11068745; doi:10.1038/s41467-024-47981-3)
Supplement: Supplementary file 3 — Reporting Summary [file 41467_2024_47981_MOESM3_ESM.pdf]

nature portfolio

Corresponding author(s): Eliane Piaggio
Jimena Tosello

Last updated by author(s): Feb 23, 2024

Reporting Summary

Nature Portfolio wishes to improve the reproducibility of the work that we publish. This form provides structure for consistency and transparency in reporting. For further information on Nature Portfolio policies, see our Editorial Policies and the Editorial Policy Checklist.

Statistics

For all statistical analyses, confirm that the following items are present in the figure legend, table legend, main text, or Methods section.

n/a	Confirmed	
☐	☒	The exact sample size (n) for each experimental group/condition, given as a discrete number and unit of measurement
☐	☒	A statement on whether measurements were taken from distinct samples or whether the same sample was measured repeatedly
☐	☒	The statistical test(s) used AND whether they are one- or two-sided<br>*Only common tests should be described solely by name; describe more complex techniques in the Methods section.*
☒	☐	A description of all covariates tested
☐	☒	A description of any assumptions or corrections, such as tests of normality and adjustment for multiple comparisons
☐	☒	A full description of the statistical parameters including central tendency (e.g. means) or other basic estimates (e.g. regression coefficient) AND variation (e.g. standard deviation) or associated estimates of uncertainty (e.g. confidence intervals)
☐	☒	For null hypothesis testing, the test statistic (e.g. F, t, r) with confidence intervals, effect sizes, degrees of freedom and P value noted<br>*Give P values as exact values whenever suitable.*
☒	☐	For Bayesian analysis, information on the choice of priors and Markov chain Monte Carlo settings
☒	☐	For hierarchical and complex designs, identification of the appropriate level for tests and full reporting of outcomes
☒	☐	Estimates of effect sizes (e.g. Cohen's d, Pearson's r), indicating how they were calculated

Our web collection on statistics for biologists contains articles on many of the points above.

Software and code

Policy information about availability of computer code

Data collection	Flow cytometry data were collected on LSRFortessa (BD) instrument or ZE5 flowcytometer (BioRad) .<br>Migration imaging was performed using epifluorescence Nikon TiE microscope equipped with a cooled CCD camera (HQ2, Photometrics), using a 10X (NA=0.3) dry objective.Electron microscopy images were acquired with a digital camera Quemesa (SIS) mounted on a Tecnai Spirit transmission electron microscope (FEI Company).<br>RNA-sequencing data were collected from RNAseq: SMARTv4 (Takara) and Illumina-sequencing (Novaseq 6000 system, Illumina. RT-qPCR data were collected from qPCR: LightCycler® 480 Instrument II and LightCycler® 480 Software (Roche) (methyation assay)
Data analysis	Flow cytometry data were analyzed on FlowJo version 10 (TreeStar) and statistical analysis performed on Prism v10.1.1 (GraphPad).<br>Genomic analyses were performed using R (version 4.1.1) in which raw counts are loaded and then normalized using R package DESeq2 (version 1.34.0).<br>Migration imaging was analysed using home-made custom macros in Fiji software.

For manuscripts utilizing custom algorithms or software that are central to the research but not yet described in published literature, software must be made available to editors and reviewers. We strongly encourage code deposition in a community repository (e.g. GitHub). See the Nature Portfolio guidelines for submitting code & software for further information.

Data

Policy information about availability of data

All manuscripts must include a data availability statement. This statement should provide the following information, where applicable:

- Accession codes, unique identifiers, or web links for publicly available datasets
- A description of any restrictions on data availability
- For clinical datasets or third party data, please ensure that the statement adheres to our policy

In-house bulk-RNAseq has been deposited in GEO database with the GEO accession number: GSE229389. Tosello, Richer et al. dataset (NSCLC) has been deposited in EGA, with accession code EGAS50000000293 for scRNAseq and EGAS50000000294 for scATAC-seq. De Simone et al. dataset, the GEO accesses are GSE40419 (NSCLC) and GSE50760 (CRC), and for Nunez et al., RNAseq data have been deposited in ArrayExpress with the E-MTAB-9112 accession code.

Research involving human participants, their data, or biological material

Policy information about studies with human participants or human data. See also policy information about sex, gender (identity/presentation), and sexual orientation and race, ethnicity and racism.

Reporting on sex and gender	Human NSCLC samples were collected at the Institut Mutualiste Montsouris (Paris, France). No gender related issues are applied to the analysis.
Reporting on race, ethnicity, or other socially relevant groupings	Race, ethnicity, or any socially relevant groupings did not play a role in the recrutiment process. Patients were recruited sequentially as they approached the IMM Hospital
Population characteristics	Covariate analysis is not applicable to these studies
Recruitment	Resection material was collected during surgery, which was proven on histopathology. There was no self-selection bias in this study. Blood buffy coats from healthy donors are collected at the Etablissement Français du sang (Paris, France).
Ethics oversight	The experimental procedures are in line with the guideline of the Declaration of Helsinki using human samples are in line with the guideline of the Declaration of Helsinki and informed consents were obtained both from cancer patients (Institut Curie) and from healthy donors (Etablissement français du sang). The protocol has been approved by the Ethic Committee of Institut Curie Hospital group (CRI- DATA190154).

Note that full information on the approval of the study protocol must also be provided in the manuscript.

Field-specific reporting

Please select the one below that is the best fit for your research. If you are not sure, read the appropriate sections before making your selection.

☒ Life sciences ☐ Behavioural & social sciences ☐ Ecological, evolutionary & environmental sciences

For a reference copy of the document with all sections, see nature.com/documents/nr-reporting-summary-flat.pdf

Life sciences study design

All studies must disclose on these points even when the disclosure is negative.

Sample size	No statistical methods were used to predetermine sample size. Sample size of animal experiments was estimated on the basis of similar research reported in our previous experience. Sample size for human data was chosen according to personal experience and overall availability of samples.
Data exclusions	In the bulk RNAseq analysis, data were curated to exclude obvious contaminant cell populations. No data were excluded elsewhere throughout the manuscript.
Replication	Numbers of healthy donors tested are mentionned in the figures or the associated legend. All performed experiments are mentioned in the reporting summary
Randomization	For in vivo tumor growth experiments, tumor-bearing mice were randomized prior to the distribution in the different experimental groups to ensure equivalent tumor burden prior to treatment. Randomization is not relevant for the rest of the studies
Blinding	No blinding was used to assign and constitute experimental groups. Our readouts consist of objective measurements.

Reporting for specific materials, systems and methods

We require information from authors about some types of materials, experimental systems and methods used in many studies. Here, indicate whether each material, system or method listed is relevant to your study. If you are not sure if a list item applies to your research, read the appropriate section before selecting a response.

Materials & experimental systems

n/a	Involved in the study	
[ ]	[x]	Antibodies
[ ]	[x]	Eukaryotic cell lines
[x]	[ ]	Palaeontology and archaeology
[ ]	[x]	Animals and other organisms
[ ]	[x]	Clinical data
[x]	[ ]	Dual use research of concern
[x]	[ ]	Plants

Methods

n/a	Involved in the study	
[x]	[ ]	ChIP-seq
[ ]	[x]	Flow cytometry
[x]	[ ]	MRI-based neuroimaging

Antibodies

Antibodies used

Flow cytometry
Live/Dead Aqua Invitrogen L34957
Live/Dead NIR Invitrogen L10119
CD45 APCcy7 2D1 BD 557833
CD3 BV650 OKT3 BioLegend 317324
CD4 BV786 OKT4 Biolegend 317442
CD4 APC M-T466 Miltenyi 130-113-250
CD74 FITC 5-329 Invitrogen 11-0748-41
CD74 PEvio770 5-329 Miltenyi 130-101-506
CD74 APCvio770 5-329 Miltenyi 130-101-534
Foxp3 PEcf594 236A/E7 BD 563955
CD8 PEcf594 RPA-T8 BD 562282
CD127 FITC MB15-18C9 Miltenyi 130-113-409
CD25 PE M-A251 BD 555432
CD25 FITC M-A251 BD 555431
Foxp3 PEcf594 236A/E7 BD 563955
CD74 PEvio770 5-329 Miltenyi 130-101-506
CD74 APCvio770 5-329 Miltenyi 130-101-534
HLADR BUV805 G46-6 BD 748338
PD1 BV711 EH12.2H7 Biolegend 329928
GITR PEcy5 108-17 Biolegend 311608
CCR8 BUV395 433H BD 747573
ICOS BUV496 DX29 BD 750321
OX40 APCVio770 REA621 Miltenyi 130-127-543
OX40 APCcy7 REA621 Miltenyi 130-127-543
CTLA4 APC REA1003 Miltenyi 130-116-811
TIGIT PE REA1004 Miltenyi 130-116-814
41BB BV421 4B4-1 BD 564091
CD45 BV605 HI30 Invitrogen Q10051
HLAA2 PE BB7.2 BD 558570
CD45 BV605 HI30 Invitrogen Q10051
IFN V450 B27 BD 560371
Caspase-3 PEcy7 D3E9 cell signaling 64772S
CD44 VioBlue DB105 Miltenyi 130-113-899
CXCR4 APC 12G5 Miltenyi 130-124-017
CTV Invitrogen C34557
CFSE Invitrogen C34554
CD45.1 AF700 A20 BioLegend 110724
CD45.2 APCcy7 104 BioLegend 109824
TCRb FITC H57-597 BD 553171
CD19 BV650 6D5 BioLegend 115541
CD4 BUV737 RM4-5 BD 564933
CD8 BUV385 53-6.7 BD 563786
CD25 PEcy5 PC61.5 eBioscience 15-0251-82
Foxp3 PE FJK-16s eBioscience 12-5773-82
CD74 AF647 In1/CD74 BioLegend 151004

Immunostaining
CD74 FITC 5-329 Invitrogen 11-0748-41
HLA-DR BV711 L243 Biolegend 307644

	Phalloidin AF546 - Thermo Fisher A22283<br>EEA1 - CA5810 Cell Signalling 3288S<br>Rab6 - D37C7 Cell Signalling 9625S<br>Anti-rabbit AF568 polyclonal Thermo Fisher A11011<br>Anti-rabbit AF647 polyclonal Thermo Fisher A21245
Validation	Antibodies were all sourced commercially and titrated in house and dilutions are mentionned in the supplementary table S1. All the commercially available antibodies were validated according to the manufacturer's website

Eukaryotic cell lines

Policy information about cell lines and Sex and Gender in Research

Cell line source(s)	B16-F10 melanoma tumor cell line (CRL-6475) and HCC827 human lung (female) tumor cell line (CRL-2868) were obtained from the ATCC. MCA101 fibrosarcoma was kindly given by Clotilde Thery and MDA-MB231 (female), MCF-7 (female) by Pascale Hubert (Institut Curie, Paris, France), MC38 by Nicole Haynes (Peter MacCallum Cancer Centre, Melbourne, Australia).
Authentication	HCC827 and MDA-MB231 were authenticated using Short Tandem Repeat test.
Mycoplasma contamination	These cell lines were negative for mycoplasma contamination as detected by PCR.
Commonly misidentified lines (See ICLAC register)	No commonly misidentified cell lines were used in this study

Animals and other research organisms

Policy information about studies involving animals; ARRIVE guidelines recommended for reporting animal research, and Sex and Gender in Research

Laboratory animals	Laboratory mouse animals used in this study are described in the methods section. C57Bl/6 were purchased from Charles River (France), NSG mice (NOD.Cg-Prkdcscid Il2rgtm1Wjl/SzJ, JAX#005557) are from Charles River (France), Bl/6 Rag2-/- KP as a source of Rag2-/- mice are bred in house, CD45.2 Cd74-/- C57Bl/6 mice were a gift from A.M. Lennon, Institut Curie, France. CD74fl/flFoxp3eGFP-Cre-ERT2 were generated from CD74fl/fl obtained by the CIPHE (Marseille, France) crossed with Foxp3eGFP-Cre-ERT2 (JAX#016961) kindly given by Dr J. Kanellopoulos (Université Paris-Saclay, France). Mice were 2-4 months old. Experimental and control animals were co-housed with housing conditions using a 12 light/12 dark cycle, with a temperature between 20 and 24 °C with an average humidity rate between 40% and 70%. Human endpoints were used for tumor-bearing mice as maximal ethical size of tumors , more than 20% of weight loss, signs of altered mobility-eating ability and cachexia.
Wild animals	Study did not involve wild animals.
Reporting on sex	All the mice used were females, except for the CD74fl/fl/Foxp3eGFP-Cre-ERT2 for which males were used when described in the figures.
Field-collected samples	The study did not involve samples collected from field
Ethics oversight	Experimental procedures were specifically approved by the ethics committee from Institut Curie, officially registered as CEEA-IC #118 and the Ministère de l'enseignement supérieur, de la recherche et de l'innovation which validated the project with the reference (APAFIS#39042-2022103116127877 v2) in compliance with the international guidelines.

Note that full information on the approval of the study protocol must also be provided in the manuscript.

Clinical data

Policy information about clinical studies

All manuscripts should comply with the ICMJEguidelines for publication of clinical research and a completedCONSORT checklist must be included with all submissions.

Clinical trial registration	*Provide the trial registration number from ClinicalTrials.gov or an equivalent agency.*
Study protocol	*Note where the full trial protocol can be accessed OR if not available, explain why.*
Data collection	*Describe the settings and locales of data collection, noting the time periods of recruitment and data collection.*
Outcomes	*Describe how you pre-defined primary and secondary outcome measures and how you assessed these measures.*

Plants

Seed stocks

Report on the source of all seed stocks or other plant material used. If applicable, state the seed stock centre and catalogue number. If plant specimens were collected from the field, describe the collection location, date and sampling procedures.

Novel plant genotypes

Describe the methods by which all novel plant genotypes were produced. This includes those generated by transgenic approaches, gene editing, chemical/radiation-based mutagenesis and hybridization. For transgenic lines, describe the transformation method, the number of independent lines analyzed and the generation upon which experiments were performed. For gene-edited lines, describe the editor used, the endogenous sequence targeted for editing, the targeting guide RNA sequence (if applicable) and how the editor was applied.

Authentication

Describe any authentication procedures for each seed stock used or novel genotype generated. Describe any experiments used to assess the effect of a mutation and, where applicable, how potential secondary effects (e.g. second site T-DNA insertions, mosiacism, off-target gene editing) were examined.

Flow Cytometry

Plots

Confirm that:

☒ The axis labels state the marker and fluorochrome used (e.g. CD4-FITC).

☒ The axis scales are clearly visible. Include numbers along axes only for bottom left plot of group (a 'group' is an analysis of identical markers).

☒ All plots are contour plots with outliers or pseudocolor plots.

☒ A numerical value for number of cells or percentage (with statistics) is provided.

Methodology

Sample preparation

Human samples (tumor or lymphn node) are cut in small pieces and digested with Liberase TL (0.1 mg/ml, Roche) and DNase (0.1 mg/ml, Roche) for 30 min at 37°C with agitation. The cell suspension is recovered in CO2-independent medium (GIBCO) with 0.4% bovine serum albumin (BSA) and filtered in a 40µm- cell strainer (BD). Cells are stained with a viability marker in PBS during 15 minutes at 4°C. Following this step, they are stained with a mix of fluorochrome-conjugated antibodies against surface protein in staining buffer (PBS, 2mM EDTA, 0.5% BSA) during 20 minutes at 4°C. After two washes with staining buffer, cells are fixed in Fixation/Permeabilization kit during 30 minutes at 4°C (ThermoFisher scientific – Cat#15151976) and washed twice with Permeabilization Buffer (ThermoFisher – Cat# 12766048). Intracellular staining is done by diluting the fluorochrome -conjugated antibodies in the Permeabilization Buffer during 20 minutes at room temperature protected from light. Cells are washed twice with staining buffer and analyzed on a flowcytometer.

Instrument

LSRFortessa (BD)
ZE5 flowcytometer (BioRad)

Software

On the flowcytometer cells are acquired using FacsDIVA software and then deeply analyed with FlowJo software (v10.10 (TreeStar).

Cell population abundance

Describe the abundance of the relevant cell populations within post-sort fractions, providing details on the purity of the samples and how it was determined.

Gating strategy

Gating strategies are shown in the appropriate figures, especially in Figure supplem.S1C.

☒ Tick this box to confirm that a figure exemplifying the gating strategy is provided in the Supplementary Information.
